# Facial Emotion Recognition in Verbal Communication Based on Deep Learning

**DOI:** 10.3390/s22166105

**Published:** 2022-08-16

**Authors:** Mohammed F. Alsharekh

**Affiliations:** Department of Electrical Engineering, Unaizah College of Engineering, Qassim University, Unaizah 56452, Saudi Arabia; m.alsharekh@qu.edu.sa

**Keywords:** deep learning, facial expression recognition, law enforcement, smart cities, smart security, CNN, verbal communication

## Abstract

Facial emotion recognition from facial images is considered a challenging task due to the unpredictable nature of human facial expressions. The current literature on emotion classification has achieved high performance over deep learning (DL)-based models. However, the issue of performance degradation occurs in these models due to the poor selection of layers in the convolutional neural network (CNN) model. To address this issue, we propose an efficient DL technique using a CNN model to classify emotions from facial images. The proposed algorithm is an improved network architecture of its kind developed to process aggregated expressions produced by the Viola–Jones (VJ) face detector. The internal architecture of the proposed model was finalised after performing a set of experiments to determine the optimal model. The results of this work were generated through subjective and objective performance. An analysis of the results presented herein establishes the reliability of each type of emotion, along with its intensity and classification. The proposed model is benchmarked against state-of-the-art techniques and evaluated on the FER-2013, CK+, and KDEF datasets. The utility of these findings lies in their application by law-enforcing bodies in smart cities.

## 1. Introduction

Facial expressions play a very significant role in nonverbal communication. Nonverbal cues can be categorised as facial expressions of a non-communicative nature. It is natural and reflects not only emotions, but also several mental activities, physical gestures, and social interactions [1]. Facial expression recognition (FER) is widely used in several applications, including customer satisfaction recognition, human–computer interaction, medical diagnostics (disease), elderly care, criminal justice systems, security monitoring, smart card applications, and increased law enforcement services in smart cities [2,3,4]. Vision sensor-based FER has attracted attention in current research and has great potential in real-time FER recognition. In vision-based FER, the researchers focused on seven basic expressions, i.e., anger, disgust, fear, happy, neutral, sad, and surprise [5,6], and they categorised the FER into two sub-categories as conventional and deep learning (DL)-based methods.

Conventional FER systems comprise three major steps: face detection, feature extraction, and classification [7]. Several researchers have used conventional feature extraction mechanisms based on clustering methods [8], such as Local Binary Patterns (LBP) [9], principal component analysis (PCA) [10], Histogram-Oriented Gradient (HoG) [11], Oriented FAST and Rotated BRIEF (ORB) [4], feature-level fusion techniques [12], etc. Afterward, the extracted features are fed to the classifiers for classification, such as K-nearest neighbours (KNN) [13], Hidden Markov Models (HMM) [14], Support Vector Machines (SVM) [15], Decision trees, and Naïve Bayes [16]. In conventional vision-based FER, independent feature extraction and classification are the major concerns, require domain experts for prominent feature selection and classification, and are time-consuming and error-prone techniques [17], making it challenging to improve the system performance of conventional FER. Therefore, the researcher investigated DL-based strategies for FER, providing comparatively better accuracy.

Inspired by the recent performance of DL approaches, several researchers used CNN-based methods in different domains, such as fire disaster [18], time-series analysis [19,20], medical image analysis [21], video analysis [22], photovoltaics [23], sentiment analysis from text data [24], and energy management [20,25,26], and they achieved promising results. In recent years, DL-based methods have shown promising results for FER over conventional methods by blending end-to-end automatic feature extraction and classification into one step [27,28]. In particular, convolutional neural networks (CNNs) have been used in several research studies to address the limitations of conventional FER [29,30]. Therefore, the researcher used different CNN models for FER, such as the ensemble convolutional neural network (ECNN) for FER used in [31], VGG [5,32], AlexNet [33], ResNet50 [34], and Xception [35]. These methods improve the performance over conventional FER; however, the accuracy of FER needs further improvement, and the time complexity, model size, inferencing speed, and performance on unseen data restrict the system from real-world implementation; as such, an efficient and accurate model has yet to be developed.

In this paper, we propose an improved CNN-based architecture for FER and improve the performance of FER to increase its usability in several applications, such as human–computer interaction, customer reviews, and elderly care, and especially to increase law enforcement services in smart cities. We used the Viola–Jones (VJ) face detection algorithm, which was created based on considerable research into facial recognition and detection and which can segment and recognise elements such as the nose, mouth, and eyes [36]. The detected faces were passed to our proposed model for FER. The proposed model is lightweight and can be deployed over a cost-effective, resource-constrained device. The performance of the proposed model was evaluated using three benchmark datasets to check the model’s effectiveness in a real-world environment. The key contributions of our work are summarised as follows:

1. We propose an efficient framework for FER that can be deployed over resource-constrained hardware to identify and monitor suspicious activities that can assist law enforcement agencies in smart cities by providing a cost-effective solution to ensure better security.
2. The proposed framework is based on a lightweight CNN model for FER and uses the VJ algorithm for face detection.
3. We performed cross-validation of the proposed model to fully assess its generalisation abilities.
4. The performance of the proposed model is evaluated on several benchmark datasets and the results reveal significant improvements in accuracy compared to state-of-the-art approaches.

The rest of the work is presented as follows: Section 2 describes related work, Section 3 explains the methodology, Section 4 presents the results, and Section 5 concludes the paper.

## 2. Related Work

FER is currently the subject of considerable active research, and several cutting-edge techniques have been proposed over the past two decades. However, due to the infinitely varied level of facial features in people of different ages, cultures, genders, scales, and perspectives, the procedure requires techniques of better accuracy. The relevant literature includes numerous studies on the use of facial expressions to identify feelings and emotions. Several researchers have proposed different techniques based on conventional and DL-based methods. However, conventional vision-based FER methods have achieved limited performance in extracting features from the given input images and classifying them accordingly. For instance, Kumar et al. [8] proposed a three-tier framework for FER. In the first tier, Otsu’s thresholding approach is used to remove the background using the YCbCr colour scheme; in the second tier, the max–min algorithms are used to select the initial cluster values of K-means algorithms to segment the most important regions from the image nose, mouth, forehead, eye gap, and eye; and in the third tier, different shape features are extracted from these segmented regions and fed into a two-level rule-based classifier for FER. Shan et al. [9] employed LBP and discriminant LBP features for the given input images and fed them to the SVM classifier for effective FER. Mansour et al. applied a PCA-based method for efficient FER [10], while Kumar et al. [11] presented a real-time system for FER. The authors used the HoG features descriptor to extract the most prominent facial features and the SVM classifier to differentiate the extracted features into seven different emotions. Sajjad et al. [4] developed an FER-based system for detecting suspicious activity. The authors used the VJ algorithm for face detection; the detected face was then preprocessed by the median and Gabor filters. The ORB features were then extracted and the SVM classifier was trained to classify the seven basic emotions. Wang et al. [14] used geometric LBP and Gabor feature descriptors for salient facial feature extraction; the extracted features were then classified by HMM. Abdulrahman et al. [15] employed the PCA and LBP feature extraction mechanism, using SVM as a classifier to differentiate the extracted features in the seven basic emotions. They used a VJ algorithm for face detection, a Supervised Descent Method to extract prominent features from the detected face, and a decision tree algorithm to classify the seven basic emotions. In conventional vision-based FER, independent feature extraction and classification are the major concerns, require domain experts for prominent feature selection and classification, and are time-consuming and error-prone techniques.

DL-based methods are applied to overcome the challenges of a conventional vision-based system for FER. Numerous research studies have been done to examine FER, and some of the most recent work has focused on developing an effective and efficient training model. For instance, Mayya et al. [37] presented an approach for FER using DCNN features. The authors employed a pretrained DCNN model, which used the pretrained weight of ImageNet [38], and obtained a 9216-dimensional vector for validation with SVM to recognise the seven basic emotions. Sajjad et al. [30] proposed an FER method for behaviour analysis by considering some serious famous TV videos. In this approach, the VJ algorithm is used for face detection, and then Hog, SVM, and a CNN model are used for features extraction and classification. Al-Shabi et al. [7] used a hybrid model for FER. They fused the features of SIFT and CNN for facial feature analysis. Yu et al. [39] investigated the performance of CNNs for FER by employing an assembly of CNNs with five convolutional layers. The authors used a stochastic pooling strategy instead of maximum pooling to achieve better performance. Jain et al. [40] proposed a new DL model containing deep residual blocks and convolution layers for accurate FER. Some DL models, such as the VGG, AlexNet, four-layer CNN, ResNet, and MobileNet, are used in [5,27,33,34,41,42] for accurate FER. However, the time complexity, model size, inferencing speed, and performance on unseen data restrict these systems from real-world implementation; therefore, an efficient and accurate model has yet to be developed. The framework proposed in this paper takes advantage of techniques used to address concerns about increased computational costs and feature extraction from low-resolution images in poor-quality scenarios.

## 3. Materials and Methods

FER has become an area of interdisciplinary research. In addition to other applications, FER has a wide range of uses in the field of security, as it can be used to identify and verify a person’s impressions in a photo or video. In this work, we recognise that FER is a two-step process: (1) a live video stream using the VJ algorithm for face detection and (2) a four-layer CNN architecture for FER. 

### 3.1. Datasets

To measure and evaluate several methods of classification and recognition of facial emotions, we needed standardised datasets. Several facial emotion datasets have been developed in recent decades. The following sections present a detailed overview of some of the standard and benchmark datasets used in this work.

#### 3.1.1. FER-2013

The FER-2013 consists of 33,000 grayscale images of faces expressing the seven basic emotions of feeling neutral, happy, anger, sad, surprise, disgust, and fear [43]. Faces are automatically registered so that each image is more or less in the middle and takes up approximately the same amount of space.

#### 3.1.2. CK+

CK+ consists of 593 images of 120 people aged 18–30 years [44]. The dataset includes images that cover all seven basic emotions at a resolution of 640 × 490 or 640 × 480, in 8-bit grayscale. Approximately 81% of the people are European-American, 13% are African American, and 6% are of another ethnicity of descent, with a women-to-men ratio of 65 and 35.

#### 3.1.3. The KDEF

The KDEF [45] consists of 490 JPEG images of 35 women and 35 men depicting seven different emotional expressions at a resolution of 72 × 72 pixels. 

### 3.2. Facial Detection Using the VJ Algorithm

**Figure 1:**
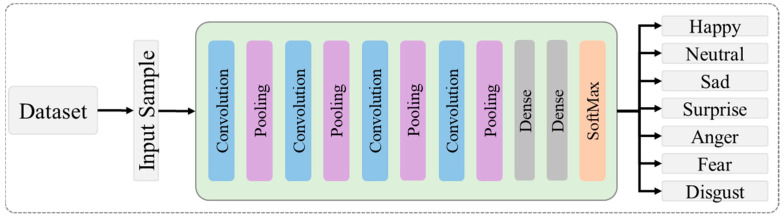
A detailed view of the proposed facial recognition model.

The VJ algorithms include the Haar feature selection, AdaBoost learning, and cascading classifier construction. The Haar features are used to recognise darker regions of the eyes from the brighter regions of the nose. This is described by comparing the pixel values of both regions and subtracting the number of estimated pixels in the bright and dark regions to find the difference. The difference is measured with a specific threshold to check the appearance of the object in the image and to classify them as nose, eyes, and chin on face or no-face. In the detection process, each detector consists of a combination of strong and weak successive classifiers, and in our case, the strong classifier is trained using AdaBoost learning through weak classifier combinations obtained by the Haar features of edge, line, or four-sided structures. The Haar features enable the process of interpreting identifiably different parts of a face by creating classifier cascades through the use of whether the identifiable portion is an edge, line, or four-sided structure. In the proposed technique, we have integrated the VJ facial detection algorithm: the camcorder captures a video, extracts the video frames as input images, crops them, and converts them into grayscale images. Once the image is converted into grayscale, it goes through the feature extraction process shown in Figure 1, which shows different images from the framework (pictures of a man, woman, and child) to detect the emotions of both genders. The proposed system is independent of the age factor. 

### 3.3. Proposed Model Architecture

In the proposed model, the faces detected by the VJ algorithm are fed into the proposed CNN architecture for prominent feature extraction and classification. The features of the selected frames are received by a series of four convolutional layers and a pooling layer, followed by the ReLu activation function. Afterward, the process frame is passed to the fully connected layer and displayed to classify the input image in its corresponding seven emotions. The proposed architecture is presented in Figure 1 and is chosen because of its speed and accuracy, and above all, it is the most reported work. In this architecture, 32 different kernels (size 3 × 3) are applied with batch normalisation and the ReLu activation function using a 224 × 224 × 3 input shape for RGB data and a 224 × 224 × 1 input shape for grayscale. We used maximum pooling with a 2 × 2 kernel size to reduce the dimensions. This process was repeated for all the remaining convolutional and pooling layers by increasing the number of kernels from 32 to 64 in the second layer, from 64 to 128 in the third layer, and from 128 to 256 in the fourth layer. In fully connected layers, 64 and 128 neurons of the first and second fully connected layers are selected, respectively, and the SoftMax layer consists of seven neurons that provide the probability of each class. Table 1 shows the internal architecture of the proposed model.

### 3.4. Flowchart Diagram of the Proposed Work

**Figure 2:**
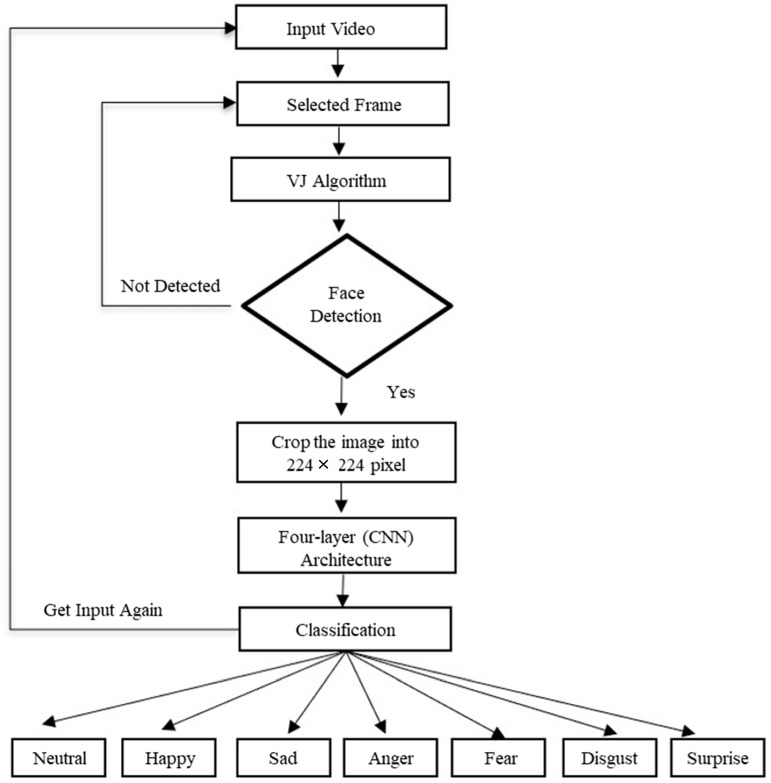
Flowchart diagram of the proposed work.

Figure 2 shows the workflow of the proposed model. The input frame is extracted from the video and each face is detected from the input image through the VJ algorithm. If the face is not detected, the system will scan another face detection video frame and the process continues until the face is detected. When the face is detected, it is cropped into a 224 × 224 pixel size and passed to the CNN model for efficient emotion recognition into the seven different classes—anger, disgust, fear, happy, neutral, sad, and surprise—for the display of the output results.

## 4. Results and Discussion

Experimental results were obtained from three benchmark datasets: FER-2013, CK+, and KDEF. The datasets were divided into training, testing, and validation data, where 60% of data were selected for training, 20% for testing, and 20% for model validation. We followed a state-of-the-art method to split the dataset between training, testing, and validation [46]. Before choosing these percentages, we also tested the proposed model over several variants of data splitting, meaning that the proposed model could effectively learn with a lower amount of data. We conducted experiments during the training and testing processes to determine the dramatic changes that occurred in the performance of the proposed system. To obtain streams from VSN [22] for experimental evaluation, we used Python 3.64, OpenCV3+, Keras, and TensorFlow with resource-constrained devices. We used a GTX 1070 GPU with 8 GB of onboard memory and an intel Core i5 processor with 16 GB of RAM to train the model on a system comprising 8 GB of memory, a 2.8 GHz processor, and a 1 terabyte (TB) installed hard drive. Table 2 shows the detailed specifications of the system and important libraries.

### 4.1. Experimental Evaluation

In this section, we evaluate the performance of the proposed model over benchmark datasets. The detailed experimental results for each dataset are described in the following subsections:

#### 4.1.1. Performance Evaluation over FER-2013

**Figure 3:**
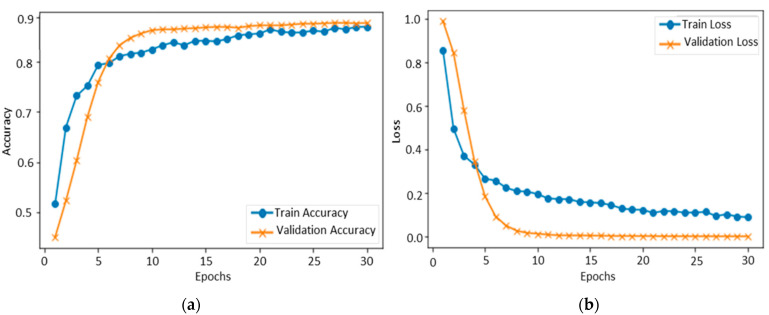
Training and validation of the proposed model over FER-2013: (**a**) accuracy and (**b**) loss.

**Figure 4:**
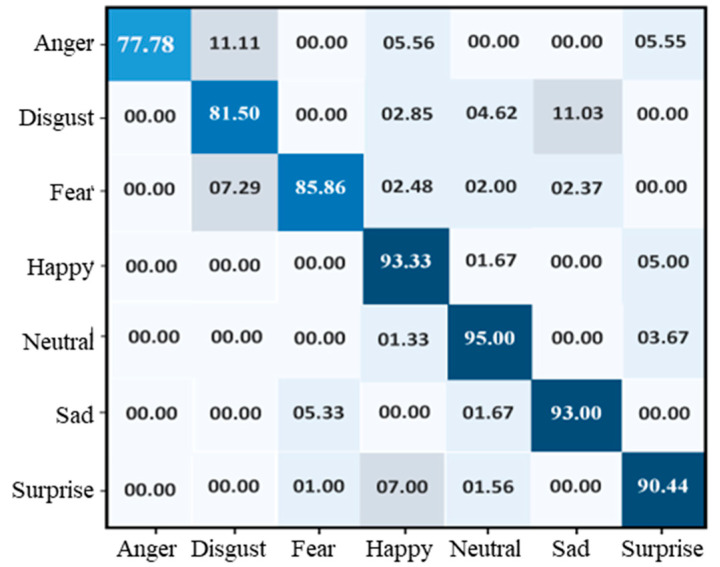
Confusion matrix of the proposed model over the FER-2013 dataset.

Figure 3 shows the accuracy level of our proposed CNN model on FER-2013 during training and validation. The x-axis shows the number of epochs, while the y-axis shows the accuracy of the proposed model concerning training and validation. We set 30 epochs as the standard for model training, and the ratio of accuracy is listed on the y-axis. The validation accuracy of the proposed method on the FER-2013 started at 0.2%, whereas the accuracy of training started at 0.5%. After each epoch, the accuracy of training and validation decreases, and after several epochs, these accuracies become stable. Over 30 epochs, the training and validation accuracy reached 89%, respectively, indicating that the proposed model fits with the data variation. The testing accuracy of each class was 77.78% for anger, 81.50% for disgust, 85.86% for fear, 93.33% for happy, 95% for neutral, 93% for sad, and 90.44% for surprise. The overall testing accuracy of our model using FER-2013 was 89%, and the confusion of all classes is given in Figure 4.

#### 4.1.2. Cross-Validation of the FER-2013 Trained Model over the KDEF and CK+ Datasets

We also evaluated the performance of the proposed model using a cross-crop evaluation matrix, where the model is trained over the FER-2013 dataset and validated over KDEF and CK+ datasets. Table 3 and Table 4 present the detailed results. Cross-crop validation was performed over a test set of data to check the generalisation ability of the proposed model over unseen data.

#### 4.1.3. Performance Evaluation over CK+

**Figure 5:**
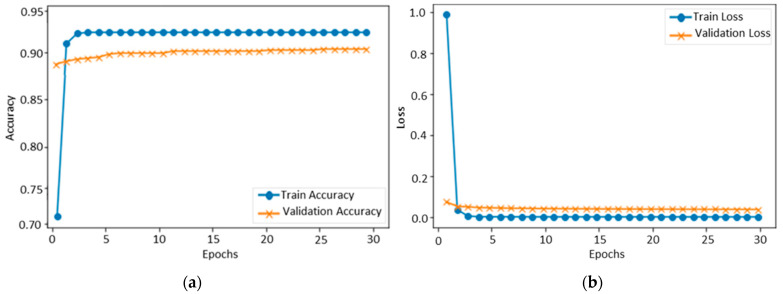
Training and validation of the proposed model over FER-2013: (**a**) accuracy and (**b**) loss.

**Figure 6:**
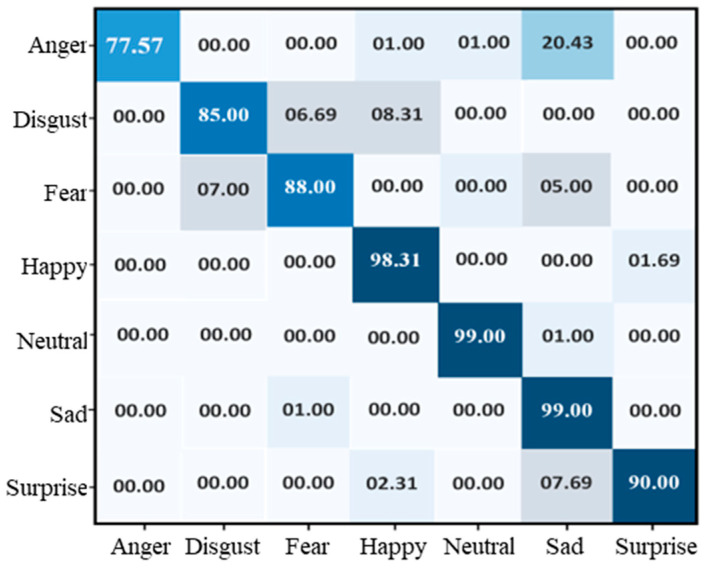
Confusion matrix of the proposed model over the CK+ dataset.

To evaluate the performance of the proposed model using the CK+ dataset, we experimented with the same number of epochs as previously used for the FER-2013 dataset. The training and accuracy of our model using the CK+ dataset rose from 71% and 90%, as shown in Figure 5, and the accuracy of training and validation reached 92% and 89%, respectively. The testing accuracy is shown in the confusion matrix, as given in Figure 6, which indicates that the overall testing accuracy of the proposed system on CK+ is 90.98%. The proposed model achieved 77.57%, 85%, 88%, 98.31%, 99%, 99%, and 90%, respectively, for the anger, disgust, fear, happy, neutral, sad, and surprise classes.

#### 4.1.4. Cross-Validation of CK+ Trained Model over FER-2013 and KDFE Dataset

The performance of the CK+ dataset-trained model was cross-validated on FER-2013 and CK+ datasets. We selected test samples from each class of the mentioned datasets and performed a cross-validation to verify the robustness of the model. Table 5 and Table 6 present the results. 

#### 4.1.5. Performance Evaluation of KDEF

**Figure 7:**
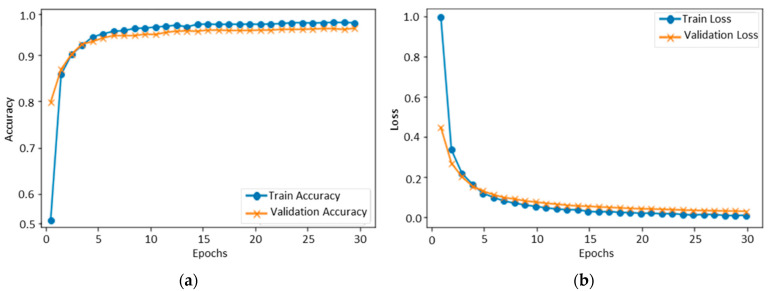
Training and validation of the proposed model over the KDEF dataset: (**a**) accuracy and (**b**) loss.

**Figure 8:**
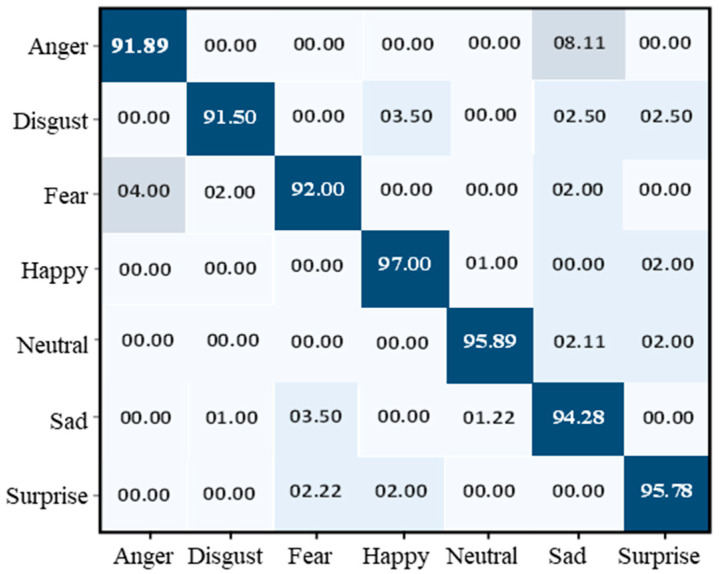
Confusion matrix of the proposed model over the KDEF dataset.

Figure 7 illustrates the results of the KDEF dataset in terms of validation and training accuracy, which started at 0.5%. After each epoch, the accuracy gradually increased because of the learning parameters programmed into the machine. Finally, over 30 epochs, the accuracy of training and validation reached 94% and 93%, respectively. The KDEF final confusion matrix indicated that the overall testing accuracy of the proposed model was 94.04%. Anger is identified at an accuracy rate of 91.89%, disgust at 91.50%, fear at 92%, happy at 97%, neutral at 95.89%, sad at 94.28 %, and surprise at 95.78%, as shown in Figure 8.

#### 4.1.6. Cross-Validation of the KDEF-Trained Model over the FER-2013 and CK+ Datasets

The trained model over the KDEF dataset was cross-validated with the FER-2013 and CK+ datasets, and the detailed results are given in Table 7 and Table 8. Cross-crop validation was performed over a test set of data to check the generalisation ability of the proposed model over unseen data.

To conclude our analysis of all the above-mentioned results, the proposed model was trained on FER-2013, CK+, and KDEF datasets individually, and their performance was validated. Furthermore, each model was cross-validated on the other two datasets to fully assess the generalisation ability of the proposed model.

Based on the above-mentioned results, the proposed model achieved remarkable accuracy over each dataset; however, the performance of the proposed model over the CK+ dataset achieved lower accuracy against the FER-2013, and the FER-2013 achieved lower accuracy against the KDEF. The performance of the proposed model over the CK+ dataset achieved lower accuracy against FER-2013 due to unbalanced samples in FER-2013 datasets when the model was trained over a balanced CK+ dataset. Furthermore, the proposed model achieved lower accuracy in cross-crop validation when the model was trained over FER-2013 and validated over KDEF. The main reason behind the lower performance of the model is that KDEF is an RGB dataset, while the FER-2013 samples were in grayscale.

### 4.2. Comparative Analysis of the Proposed Model with State-of-the-Art Techniques

We conducted several experiments to evaluate the performance of the proposed FER model with other state-of-the-art methods, as shown in Table 9. To evaluate the model’s robustness, we first compared the performance of our model with FER-2013; the proposed model surpassed an accuracy of 23.2%, 17.2%, 0.6%, and 2.42%, respectively, compared to the models of Arriaga et al. [51], J. Li et al. [52], Subramanian et al. [53], and Borgalli et al. [46]. We also assessed the robustness of our model using the CK+ dataset, where our model achieved a promising result compared to state-of-the-art methods. The proposed model achieved 1.48%, 9.98%, and 3.29% higher accuracy compared with those of Hasani et al. [54], Borgalli et al. [46], and Bodapati et al. [6], respectively. We then assessed the proposed model using the KDEF dataset. Our model obtained a higher accuracy of 0.65 when compared with the method proposed by Sajjad et al. [30]. To further analyse the model, Haq et al. [55] and Liu et al. [56] achieved 0.39% and 7.14% lower performance compared to the proposed model. 

### 4.3. Time Complexity of the Proposed Model over GPU, CPU, and Resource-Constrained Devices

We evaluated the performance of a proposed model in real time to compute the processing time of the proposed model over GPU, CPU and resource-constrained device (Jetson Nano). Jetson Nano is a small and powerful computer that runs multiple CNNs in parallel for different applications, such as recognition, segmentation, object detection, and speech processing. Its GPU has 128 NVIDIA CUDA^®^ cores, the CPU is Quad-core ARM Cortex-A57, and it has 4 GB of memory. The frames per second (fps) of the proposed model using GPU, CPU, and Jetson Nano were 45, 21, and 26 s, respectively. The time complexity of the proposed model is much lower and applicable for deployment in real-world scenarios.

## 5. Conclusions

The capabilities built into FER technology with resource-constrained devices, such as the Jetson Nano, can greatly assist law enforcement agencies in effectively identifying suspects by analysing a person’s facial expressions. This requires an effective framework to facilitate the identification of fake and suspected individuals from facial expressions. With this in mind, we have proposed an efficient facial expression framework using Jetson Nano, a resource-limited tool that measures facial expressions from video streams captured by the VSN. The proposed framework automatically extracts the face using the VJ algorithm and then identifies facial expressions using the proposed model. The proposed model achieved significantly better results compared to the other methods. The quantitative and qualitative capacities using three different datasets demonstrated the effectiveness of the proposed framework for enhancing law enforcement services in smart cities. In future studies, we will extend the proposed framework to incorporate gender classification and age-predicting factors for the identification of facial emotions in detail. Such a system would enable us to determine the gender, age, and emotions of individuals effectively. We will apply various DL models and review their performance on resource-constrained devices. We will also apply data augmentation techniques to balance the samples in each class and increase the number of samples for all classes to further improve the performance of the proposed model.

## Figures and Tables

**Table 1:** The internal architecture of the proposed model.

Layer	Output-Shape	Params	Layer	Output-Shape	Params
conv2d_63 (Conv2D)	(56 × 56 × 32)	896	batch_normalisation_64	(23 × 23 × 128)	512
activation_63 (Activation)	(56 × 56 × 32)	0	conv2d_66 (Conv2D)	(21 × 21 × 256)	295,168
max_pooling2d_32	(28 × 28 × 32)	0	activation_66 (Activation)	(21 × 21 × 256)	0
batch_normalisation_63	(28 × 28 × 32)	128	batch_normalisation_66	(21 × 21 × 256)	1024
conv2d_64 (Conv2D)	(26 × 26 × 64)	18,496	flatten_16 (Flatten)	(112,896)	0
activation_64 (Activation)	(26 × 26 × 64)	0	dense_42 (Dense)	(64)	7,225,408
max_pooling2d_33	(25 × 25 × 64)	0	dropout_27 (Dropout)	(64)	0
batch_normalisation_64	(25 × 25 × 64)	256	dense_43 (Dense)	(128)	8320
conv2d_65 (Conv2D)	(23 × 23 × 128)	73,856	dropout_28 (Dropout)	(128)	0
activation_65 (Activation)	(23 × 23 × 128)	0	dense_44 (Dense)	(7)	903
Total params			7,624,967		

**Table 2:** Software specifications for model training.

Name	Configuration
OS	Window 10.
Programming Language	Python 3.6 [47].
Libraries	Keras, TensorFlow, Numpy, PyLab, pillow, lxml, Cython, pandas, Matplotlib [48].
Imaging Libraries	OpenCV 3.4.0 [49], and Scikit-Learn.
Performance [50]	Line Profiler, ContentBox, CommandBox, BCMStat, and CFML.
IDE	Jupyter Notebook, and Python.

**Table 3:** Cross-validation over the KDEF dataset.

	Anger	Disgust	Fear	Happy	Neutral	Sad	Surprise	Per-Class Accuracy
**Anger**	**32**	8	6	1	2	3	1	0.60
**Disgust**	3	**52**	4	6	1	2	1	0.75
**Fear**	1	5	**31**	0	1	5	2	0.69
**Happy**	0	1	0	**61**	0	0	6	0.90
**Neutral**	2	3	2	1	**54**	3	3	0.79
**Sad**	1	1	2	0	1	**43**	0	0.90
**Surprise**	0	1	1	2	3	1	**32**	0.80
Overall accuracy								**0.78**

**Table 4:** Cross-validation over CK+ dataset.

	Anger	Disgust	Fear	Happy	Neutral	Sad	Surprise	Per-Class Accuracy
**Anger**	**185**	26	17	0	5	16	6	0.72
**Disgust**	10	**220**	29	7	15	11	3	0.74
**Fear**	7	10	**99**	0	0	8	0	0.80
**Happy**	1	5	1	**310**	0	1	27	0.90
**Neutral**	1	3	1	0	**83**	2	0	0.92
**Sad**	4	6	3	0	1	**126**	0	0.90
**Surprise**	1	4	2	22	2	5	**378**	0.91
Overall accuracy								**0.84**

**Table 5:** Cross-validation over the FER-2013 dataset.

	Anger	Disgust	Fear	Happy	Neutral	Sad	Surprise	Per-Class Accuracy
**Anger**	**574**	96	193	0	0	95	0	0.60
**Disgust**	7	**56**	11	0	24	11	0	0.51
**Fear**	4	112	**614**	0	204	90	0	0.60
**Happy**	0	177	0	**1419**	0	0	178	0.80
**Neutral**	0	0	85	0	**986**	112	50	0.80
**Sad**	65	83	130	0	97	**872**	0	0.70
**Surprise**	0	0	92	10	64	0	**665**	0.80
Overall accuracy								**0.68**

**Table 6:** Cross-validation over the KDEF dataset.

	Anger	Disgust	Fear	Happy	Neutral	Sad	Surprise	Per-Class Accuracy
**Anger**	**42**	5	2	1	0	1	2	0.79
**Disgust**	3	**48**	9	2	1	4	3	0.68
**Fear**	0	1	**39**	0	1	3	1	0.87
**Happy**	0	1	0	**61**	1	0	5	0.90
**Neutral**	1	2	1	0	**61**	2	1	0.90
**Sad**	1	2	1	0	1	**43**	0	0.89
**Surprise**	0	0	1	1	1	1	**36**	0.90
Overall accuracy								**0.85**

**Table 7:** Cross-validation over the FER-2013 dataset.

	Anger	Disgust	Fear	Happy	Neutral	Sad	Surprise	Per-Class Accuracy
**Anger**	**862**	10	86	0	0	0	0	0.90
**Disgust**	2	**89**	8	0	9	3	0	0.80
**Fear**	9	17	**901**	0	12	85	0	0.87
**Happy**	0	2	5	**1738**	3	0	29	0.98
**Neutral**	23	44	15	9	**1085**	39	18	0.88
**Sad**	36	55	97	0	0	**1059**	0	0.85
**Surprise**	0	11	9	63	0	0	**748**	0.90
Overall accuracy								**0.88**

**Table 8:** Cross-validation over the CK+ dataset.

	Anger	Disgust	Fear	Happy	Neutral	Sad	Surprise	Per-Class Accuracy
**Anger**	**180**	26	22	0	8	13	6	0.70
**Disgust**	13	**187**	39	11	23	17	5	0.63
**Fear**	16	23	**68**	1	3	11	2	0.54
**Happy**	2	6	1	**301**	0	2	33	0.87
**Neutral**	2	3	1	0	**81**	2	1	0.90
**Sad**	8	14	8	1	3	**105**	1	0.75
**Surprise**	5	6	4	45	6	9	**340**	0.81
Overall accuracy								**0.74**

**Table 9:** Comparing the performance of the proposed model with the state-of-the-art method over three benchmark datasets.

Dataset	References	Methods	Average Accuracy (%)
**FER-2013**	Arriaga et al. [51]	Mini-Xception	66.0
	J. Li et al. [52]	CNN with Transfer Learning	72.0
	Subramanian et al. [53]	Three Layer CNN architecture	88.6
	Borgalli et al. [46]	Six Layer CNN architecture	86.78
	The Proposed model	Proposed	**89.2**
**CK+**	Borgalli et al. [46]	Six Layer CNN architecture	81.0
	Bodapati et al. [55]	InceptionResNetV2	87.69
	The Proposed model	Proposed	**90.98**
**KDEF**	Sajjad et al. [30]	Fine-tuned AlexNet	93.39
	Haq et al. [55]	CNN with Transfer Learning	93.65
	Liu et al. [56]	Multi-channel features	86.9
	The Proposed model	Proposed	**94.04**

## Data Availability

Not applicable.
